# A Study on Thermal and Nanomechanical Performance of Cellulose Nanomaterials (CNs)

**DOI:** 10.3390/ma10070718

**Published:** 2017-06-28

**Authors:** Nadir Yildirim, Stephen Shaler

**Affiliations:** 1 Forest Industry Engineering, Bursa Technical University, Bursa 16310, Turkey; 2 School of Forest Resources, University of Maine, Orono, ME 04469-5755, USA; shaler@maine.edu; 3 Advanced Structures & Composites Center, University of Maine, Orono, ME 04469, USA

**Keywords:** cellulose nanomaterials (CNs), cellulose nanofibrils (CNFs), cellulose nanocrystals (CNCs), atomic force microscope (AFM), nanoindentation (NI), nanomechanical properties, thermal stability, oliver-pharr approach, fused silica approach

## Abstract

Wood-based cellulose nanomaterials (CNs) (specifically, cellulose nanofibrils (CNFs) and cellulose nanocrystals (CNCs)) are environmentally sourced low-impact materials with remarkable thermal, mechanical, and physical properties. This uniqueness makes them great candidates for creating nanocomposite materials with a wide range of attributes. Investigating the morphological, thermal, and nanomechanical properties of CNs becomes crucial to intelligent development of novel composite materials. An atomic force microscope equipped with a nanoindenter was used to investigate the compression modulus of CNFs and CNCs using two analytical approaches (denoted as Oliver Pharr (OP) and Fused Silica (FS)). The CNC modulus values (E_CNC-FS_ = 21.1 GPa, E_CNC-OP_ = 28.7 GPa) were statistically larger than those obtained from CNFs (E_CNF-FS_ = 12.4 GPa, E_CNF-OP_ = 15.1 GPa). Additionally, the FS analytical approach provided statistically significant lower estimates. Thermal stability of CNFs and CNCs was investigated using thermogravimetric analysis. Significant differences were found between CNF and CNC onset temperatures (Onset_CNC_ = 228.2 °C, Onset_CNF_ = 279.9 °C), decomposition temperatures (DTGA_CNC_ = 247.9 °C, DTGA_CNF_ = 331.4 °C), and residues (Residue_CNC_ = 34.4%, Residue_CNF_ = 22.8%). This research enriches the information on thermal stability and nanomechanical performance of cellulose nanomaterials, and provides increased knowledge on understanding the effect of CNs as a matrix or reinforce in composites.

## 1. Introduction

Reducing energy consumption, decreasing the dependency on unstable and uncertain petroleum sources, and an increase in environemental awareness produced and motivated researchers to work on developing new products using bio-based, eco-friendly, and sustainable polymers. Due to it’s nature, wood and its components are great candidates to develop ecofriendly products, since they have a very low environmental impact. Wood is an abundant renewable raw material, consisting of cellulose, hemicelluloses, lignin, and extractives in its structure. A crucial compound—cellulose—can be found as individual fibrils or as bundles in the cell walls, “glued” together by hemicelluloses and lignin [1]. Cellulose nanomaterials (CNs) are the nanoscaled structure of celluloses that can be obtained from wood, plants, bacterial cellulose, and even sea animals [2]. These celluloses are renewable, eco-friendly nanomaterials with outstanding mechanical and thermal properties. Their outstanding properties make CNs a perfect candidate as reinforcement materials to be used in composite structures. CNs can be produced by breaking wood down into nanometer-scale fibrils and particles, and can be used for unique applications [3]. CNs can be categorized into three types: cellulose nanofibrils (CNFs), cellulose nanocrystals (CNCs), and bacterial celluloses (BCs) [4]. In this study, one of the starting materials was softwood CNFs, which is referred to in literature as nanofibrillated cellulose (NFC), microfibrillated cellulose (MFC), or cellulose microfibrils (CMF). The second starting material was cellulose nanocrystals (CNCs), also referred to as micro crystalline cellulose (MCC), nano crystalline cellulose (NCC), or cellulose nanowhiskers (CNWs) in the literature. The abbreviations used in this study are CNFs for cellulose nanofibrils and CNCs for cellulose nanocrystals.

The overall goal of this study is to provide introductory information to academic and industrial researchers working on developing composite materials using nanocellulose. Knowledge of the mechanical performance of nanoscaled materials is crucial to the development of new composites. Additionally, the thermal stability of reinforcements is required to select appropriate processing and use conditions.

The lack of information on the nanomechanical performances of cellulose nanomaterials motivated many researchers to work on investigating the nanomechanical properties of CNs. Nanoindentation is a well established method to determine the mechanical performances of nanomaterials. A recent study on determining the mechanical performances of microcrystalline cellulose and investigating the effect of acid hydrolysis using nanoindentation provided modulus values 3.5–6 GPa for MCC [5]. Other research on cellulose nanocrystals’ dynamic mechanical and nanoindentation behavior showed that CNCs have modulus values that vary between 5 GPa and 9 GPa, where the differences can be related to different source of nanocelluloses, particle sizes, and the testing conditions. In the same study researchers mentioned that adding CNCs increases the mechanical performance of the composite material [6]. The study on investigating the size effect on the mechanical performances of nanocellulose, using the atomic force microscope (AFM) technique, showed that when CNC diameter is decreased the *E*_t_ (transverse elastic modulus) is increased, which was found 17.7 ± 5 GPa [7]. Due to differing values from previous studies, additional information on the nanomechancial performance of the CNs is required and more research will provide better and more accurate results for future investigations. Thermal stability is another important factor which plays a critical role in developing new formulations for creating new composites. A study showed that cellulose nanofibrils with 5–40 nm start thermal decomposition at 310 °C, and persist until 400 °C [8]. Another study on creating new composite, using nanocellulose as reinforce material, showed that addition of 1, 3 and 5% wt nanocellulose reinforces the thermal stability of final product.

This study measured the nanomechanical behavior and thermal stability of two classes of cellulose nanomaterials (CNFs and CNCs). An Asylum Research MFP-3D Atomic force microscope, (AFM) equipped with nanoindenter (NI) and MFP Instrumented Nanoindenter^TM^, was used to investigate the nanomechanical performance of CNFs and CNCs. The nanoindentation behavior was analyzed using two different approaches; the Oliver-Pharr (OP) [9] equation and a Fused Silica (FS) calibration method. The thermal stabilities of CNFs and CNCs were determined from 20 °C to 500 °C, with a 10 °C increase per minute using a TA Instruments Q500.

## 2. Materials and Methods

In this study two types of cellulose nanomaterials (cellulose nanofibirls and cellulose nanocrystals) were used. The cellulose nanofibrils (3.0–4.0 w% aqueous gel) and cellulose nanocrystals (11.5–12.5 w% aqueous gel) were obtained from The Process Development Center (PDC), University of Maine. The CNFs were produced applying mechanical forces, using the double disk refiner system. The CNCs used in this study were produced using sulfuric acid hydrolysis to break down the amorphous regions in the structure, and to leave the crystalline cellulose to have a uniform, well designed structure with high crystallinity.

### Sample Preparation

Starting materials: CNF/water suspensions were obtained at 3.4% solids by weight, and CNC/water suspension was obtained at 11.9% solids by weight. The solid contents of the suspensions were determined using a moisture content analyzer (Sartorius Precision MA-37, Sartorius AG, Goettingen, Germany) before preparing the samples. The samples (films in this case) need to have smooth surfaces for AFM and NI applications. Film thickness has been shown to significantly impact nanomechanical response. Hay and Crawford [10] investigated the substrate effect on nanomechanical measurements, and showed that stiff films need to have an indentation depth (*h*) to film thickness (*t*) ratio (*h/t*) equal or smaller than 25%, to minimize the errors in nanomechanical measurements that occur due to the substrate effect. For this purpose, CNF and CNC suspensions were dripped on the aluminum stubs with thicknesses varying from 8 μm to 15 μm, using partial oven drying (5 min at 40 °C) and air drying (till completion of the drying at 23 °C). The *h/t* ratio used in this research was kept under 5%.

## 3. Morphology

The morphologies (fibril and whisker distributions, directions, and diameters) of CNFs and CNCs films were examined using an Asylum Research MFP-3D atomic force microscope (AFM, Oxford Instruments, Goleta, CA, USA). For imaging, tapping mode is as known as non-contact or AC mode, and was performed using an Asylum Research AC240TS-10 cantilever tip with a 9 ± 2 nm radius (with spring constant, k (N/m) = 2 × (0.5–4.4)).

## 4. Nanoindentation 

The previously developed sampling method by Yildirim and Shaler (2016) was applied for each sample. For increased accuracy, 64 nanoindents (8 × 8 array over a 20 μm × 20 μm scan area) were applied to each group (calibration, and actual CNF and CNC samples) for nanoindentations. (NI-MFP Instrumented Nanoindenter™, Oxford Instruments, Goleta, CA, USA)

The calibration is a very critical step for measurements in nanoscale. For the evaluations, Oliver-Pharr [9] equations were used. However, a critical factor in the calculations (the contact area (*A*))—which goes into the equations directly—was calculated through two (2) different approaches. The approaches used in this research were Oliver Pharr (OP) and Fused Silica (FS).

### 4.1. Oliver Pharr (OP) Approach

In the OP approach, the projected area (*A*) created on the surface after nanoindentation was assumed equal to the perfect Berkovich tip geometrical area (1). This is a strong assumption, which not only assumes that the area of the tip has a flawless geometrical Berkovich shape, but also assumes that the created area on the film surface will be identical to the tip’s geometrical area. The mathematical area calculation of the Berkovich shape, which was used for the OP approach area calculations, is given below (1).

(1) $$A=24.5\times H^{2},$$

where: *H* = depth of indentation.

### 4.2. Fused Silica (FS) Approach

In the FS approach, a fused silica quartz (FSQ) specimen with 68 GPa–72 GPa [9] actual modulus value was nanoindented using an Asylum Research Nanoindenter (MFP-3D Atomic Force Microscope), fitted with a diamond Berkovich tip (Micro Star Technologies, Huntsville, AL, USA). The nanoindentation evaluations were performed using Oliver Pharr equations, and the obtained results were compared with the modulus value of the fused silica quartz (68 GPa–72 GPa [9]). The difference between the OP calculated modulus and the fused silica quartz actual modulus was determined, and a correction function was developed to correct the Area calculation and minimize the errors that had occurred, due to strong area calculation assumptions used in the OP Approach.

### 4.3. Nanoindentation on CNs

The nanoforces were applied to the CNs’ surfaces at a 125 μms^−1^ indentation rate. The slope of the unloading curves were used to calculate the stiffness of the samples. The stiffness value (*S*) of CNs was determined using the unloading curve slopes (n = 64), obtained from the force—indentation data.

The indented area (projected area, *A*) was calculated at 60 nm to a couple of hundred nm (*H* range) using the two different approaches previously described (OP and FS). The reduced modulus (*E*_R_) was then calculated (2) using the measured stiffness and area information from actual testing.

(2) $$E_{R}=\left(\frac{S\sqrt{\pi }}{2\sqrt{A}}\right),$$

Equation (3) was then used to calculate *E*_CNx_ (CN_x_ = CNF and CNC).

(3) $$E_{\mathrm{CNF}}=\left(\frac{1-\nu_{\mathrm{CNx}}{}^{2}}{\frac{1}{E_{R}}-\frac{1-\nu_{\mathrm{ind}}{}^{2}}{E_{\mathrm{ind}}}}\right),$$

In this study, the *E* value of the diamond Berkovich tip (*E*_ind_) and the Poisson’s ratio (*ν*_ind_) were assumed to be 865 GPa and 0.2, respectively. The Poissons ratio (*ν*_CNx_) of the sample CNs was assumed to be 0.3 [11,12,13].

## 5. Thermal Stability

The thermal stability of CN’s was determined using a TA Instrument Q 500 thermogravimetric analyzer (TA Instruments, New Castle, PA, USA). Evaluations were performed according to ASTM E1868–10. Five (5) samples from each group were evaluated with a 10 °C per minute ramp, ranging from 20 °C to 500 °C. Initial sample weights varied from 10 mg to 15 mg.

## 6. Statistical Analysis

The modulus (*E*), onset temperature, decomposition temperature (DTGA), and residue were analyzed and compared by conducting a one-way Means/ANOVA to check if there was a significant overall difference (significance level (alpha) = 0.01) due to difference in the type of cellulose nanomaterials (CNF vs. CNC). A two-way ANOVA (factorial) analysis was used on the nanomechanical data to assess whether the measured response was significantly impacted by material type or analytical method, or if an interaction effect was present. Significant differences between groups were evaluated by use of a Tukey-Kramer Honestly Significant Differences (HSD) test, with alpha = 0.05.

## 7. Results and Discussion

The overall results were detailed and discussed under the following subsections:

### 7.1. Morphological Properties of CNF and CNC 

**Figure 1:**
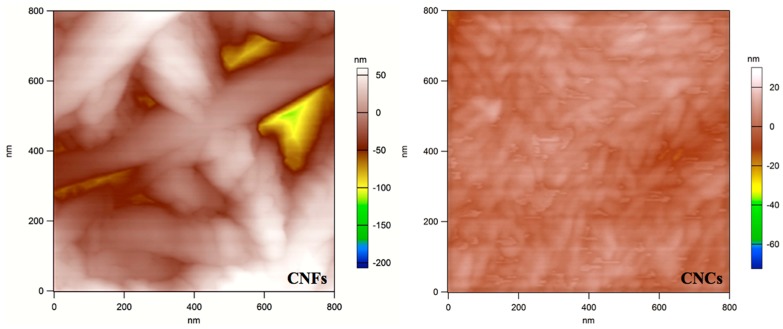
Representative AFM images of CNF and CNC samples.

Three (3) films were produced by air drying from each material. However, airdrying created imperfect (non-flat) films, due to heteregoneus drying (as expected). These non-flat samples were not suitable for imaging, so the flattest film was picked from each group and investigated. The produced CNF film included fibrils with diameters ranging between 20 and 100 nm, and lengths of the order of several micrometers. The CNC film was composed of whiskers with diameters less than 100 nm, and lengths with less than 300 nm (Figure 1).

Due to heterogenous distribution, the imperfections on the surface and high height differences in samples effected the trace-retrace quality through topogrophical imaging, and resulted in lower quality 3D images.

The nanoindentations were applied by centering the small scan grid in the filmed samples to avoid miscaluclations and evaluations. Also, researchers reviewed each force-indentation curve to see if there was skipping, cracks, and any other kind of failure through nanoindenting. Only, succesfull and accurate nanoindentations were analyzed. Results of the nanoindentations are presented and discussed in the following subsections.

### 7.2. Nanoindentations—Fused Silica

A fused silica quartz (FSQ) sample was obtained from Asylum Research for calibration. The mean modulus was calculated and statistically compared (Table 1) for two different approaches. The 64 indent stiffness values were determined to be normally distributed (Shapiro-Wilk W Test). The predicted moduli from the OP and FS area calculation procedures produced statistically significant and different results (Table 1). The estimated modulus using the OP area equation was, on average, 86% higher than that from the FS area equation.

The significant overestimation of the FSQ modulus, using the OP method, is primarily due to differences between the area of the perfect Berkovich tip and the projected area created on the sample surface.

The tip geometry changes over the course of multiple uses, due to damage and surface contamination. Due to humidity in the chamber particles, the material surface may bind to the tip, and scratches may be created through the indentation; also, potential residues can be bound through mechanical interlocking, after the indentation. This nanoscale geometry change is dependent on the medium, material property, and adhesion, etc., so it is a random change that can be limited but not controlled. Our first assumption is: the area of the tip is accepted, perfectly and geometrically, in the same way, with the area of the Berkovich shape. However, this is almost impossible after the couple measurements. This overestimation can be lowered using new tips.

A major limitation of the OP method is the assumption that the area of the Berkovich tip will be equal to the projected area created on the surface. This is mostly a material-related assumption, and brittle materials can increase overestimation, due to potential nanocracks through nanoindentation. The errors in the projected area calculations could easily increase, due this nanocracks or microcracks.

Consequently, the required correction function (a third order polynomial function $\left(A=C0H_{c}^{2}+C1H_{c}+C2H_{c}^{\frac{1}{2}}\right)$ [14]) was applied to the OP Approach results, and the accurate fused silica quartz modulus was obtained. The same correction factor, used for correcting fused silica quartz samples, applied to the CNF and CNC samples.

### 7.3. The Comparison of OP and FS Approaches Using CNF and CNC

Nanoindentations were performed on CNF and CNC films, and their nanomechanical performances were evaluated using two different approaches, to understand the importance of the chosen approach on the final result accuracy.

#### 7.3.1. Nanoindentations—CNF

A total of 64 nanoindents, created on different locations on a CNF film, were evaluated and statistically compared using OP and FS Approaches. The CNFs modulus and the average contact depth values are summarized in Table 2.

The difference between the reported modulus values in Table 2 is directly related to the area calculations. This difference can easily go up, due to the use of a contaminated tip; the imperfections on the tip will directly increase the difference in area calculations and higher area measurement will produce lower performance properties. The results showed that the OP approach produced 21.8% higher modulus values than the FS approach, which means the calculated area for OP was lower than the calculated area for the FS approach. In this case, the tip potentially had particles on it, and it created a larger area (actual area) on the surface than the geometrical Berkovich tip area. Once the correction factor is applied, the miscalculation was corrected and the modulus values were lowered and corrected according to the actual area. The statistical difference between two approaches shows the importance of using the FS area prediction for the calculation of the modulus. As mentioned in the previous paragraph, the obtained difference in modulus—21.8%—is the error related to the miscalculation of the projected area.

#### 7.3.2. Nanoindentations—CNC

64 nanoindentations were created on the CNC film samples using the same procedure as the CNF samples and represented in Table 3.

As expected, a statistical difference was also found between the two approaches for the CNCs. The OP modulus values were again found to be significantly higher than modulus values calculated using the FS area approach. The CNF modulus value differences (*E*_CNF-FS_ = 12.4 GPa, *E*_CNF-OP_ = 15.1 GPa–21.8% difference) and the CNC modulus value differences (*E*_CNC-FS_ = 21.1 GPa, *E*_CNC-OP_ = 28.7 GPa–26.5% difference) between the two approaches were compared. The error (difference) for CNCs was found to be higher than the error in the CNFs. This showed that the error percentage in the results and failure in accuracy depend on the tested materials’ characteristics, and can easily go up. This increased error provided a good example to understand the importance of using the FS approach instead of the OP approach.

### 7.4. The Comparison of Nanomechanical Performance of CNF and CNC through OP and FS Approaches

The CNFs and CNCs were nanoindented, and their nanomechanical performances were evaluated using two different approaches: to understand the difference between their nanomechanical performances; and, also, to understand the role of the chosen approach while comparing two different materials.

Understanding the behavioral differences between the CNF and CNC under the OP and FS approaches was compared using two-way anova analysis, to see if there are significant differences due to the material, approach, and material-approach interaction. 

The comparison of the nanomechanical performance of CNF and CNC was first investigated according to the OP approach. The CNC modulus (28.7 GPa {COV, % = 7.2}) was found to be almost two (2) times (47.4%) higher than CNF modulus (15.1 GPa {COV, % = 8.4}). Also, the comparisons (according to the FS approach) showed that the CNC modulus (21.1 GPa {COV, % = 6.3}) is 41.2% higher than the CNF modulus (12.4 GPa {COV, % = 8.5}). The FS approach decreases the difference (error) between the CNF and CNC modulus, by decreasing errors through corrected area calculations.

The CNF and CNC comparison, using both approaches, showed that the modulus differences in percentages (47.4% versus 41.2%) in both approaches are very similar. This finding shows that the OP approach can be used for comparison. However, it is strongly suggested to use the FS approach for investigating the properties of new materials, to obtain more accurate results.

The results indicated that the modulus values significantly (*p* < 0.0001) differ as a function of approach and material. Also, it differs as an interaction between approach and material. The effect of the approach differs according to the different type of material, and the material differs according to the different type of approach. The least square means were investigated, and CNC (on average) showed a higher response than CNF; the OP Approach showed a higher response than the FS Approach. The interaction showed that the chosen approach effect depends on the material, and also that the effect of CNC and CNF were identical.

As a result, the nanomechanical properties of CNF and CNC were investigated using two different approaches, with the coefficient of variation values less than 13.9%. In both approaches, the CNC modulus (*E*_CNC-FS_ = 21.1 GPa, *E*_CNC-OP_ = 28.7 GPa) values were found to be higher and statistically different than the CNF modulus (*E*_CNF-FS_ = 12.4 GPa, *E*_CNF-OP_ = 15.1 GPa). These high mechanical properties of cellulose nanomaterials created an attraction to use them as reinforce materials. Therefore, many studies were performed to investigate their feasibility to use as reinforce materials. A research on reinforcement of PVA (polyvinyl acetate) using NCC (nanocrystalline cellulose) showed that an addition of 1% NCC to PVA increases the MOE by 48% [15]. Similar studies related to this research (studies on the investigation of nanomechanical properties of cellulose nanomaterials) showed that microcrystalline celluloses (MCCs), extracted from cotton jute, newsprint, and the paper filter, has modulus values that vary between 1.8 GPa and 7.6 GPa, where the researchers related the difference to the crystallinity of the MCCs [16]. Another study on nanomechanical properties of wood cellulose nanomaterials showed that the wood CNF modulus values vary between 7–12 GPa, and the wood CNC modulus has higher modulus values than the CNF modulus values [17]. Also, the research performed by Yildirim et al. [14] showed that the CNFs, evaluated using the Oliver Pharr approach, have modulus values that vary between 12.4 GPa and 24.9 GPa.

As a result, higher modulus values were obtained from CNC samples, compared to CNF samples. This can be explained with the basic composite material definition. Cellulose, by itself, is a composite, where the amorphous regions of the cellulose are the matrix and the crystalline regions are the reinforce of the cellulose [18]. CNCs clearly have a higher crystalline structure (due to their production processes where the amorphous regions are broken down using chemicals), than the CNFs, which produce higher resistance to external loads.

In this study, researchers used filmed samples for the evaluations. However, responses were obtained from an individual bundled CNFs or CNCs. Another literature study, an investigation of the transverse elastic modulus of CNC films [19], also supported the results obtained in this research. They showed that the CNC films have an 8.3 GPa transverse modulus mean value, with 0.9 GPa standard deviation (which is significantly lower than the modulus values obtained in this study). It is very normal to expect lower performance from the bulk tests of CNC or CNF films, where the number of hydrogen bonds increases; and, taking a more active role in transferring the applied stress, also a potential increase in the imperfections, voids, and bonding failures in the film (and a higher moisture content effect, due to an increased number of hydroxyl groups that are produce lower performance through the bulk tests of the films).

### 7.5. The Comparison of Thermal Stability Performance of CNF and CNC

The thermal stability performances were studied using the TA Instrument Q 500 thermogravimetric analyzer. The analyses were performed according to ASTM E1868, and reported under Table 4.

**Figure 2:**
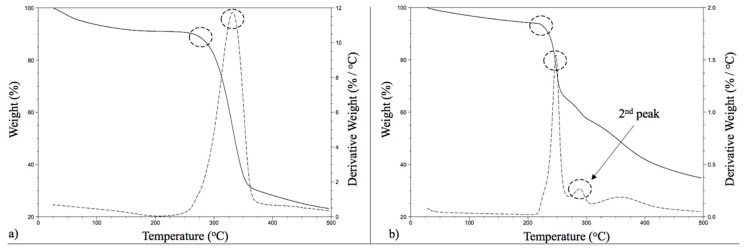
Representative TGA and DTGA curves of CNF (**a**) and CNC (**b**).

CNF and CNC are both hydrophilic polymers, due to their nature, and they keep moisture in their structure. For providing an equilibrium and stabilized moisture content, the samples were stored in laboratory conditions (23 ± 2 °C and 50% RH) for 48 h before testing. The initial weight loss, up to 10% (as shown in Figure 2), is related to moisture loss. After the weight loss related to the moisture removal, the characteristics of CNF and CNC play a role in weight loss (as a function of time and temperature).

As given in Table 4, the comparison of the thermal stability of the CNF and CNC showed that CNF has a higher onset (thermal degradation) temperature (279.9 °C) than CNC (228.2 °C). The literature studies provided parallel results with the results obtained in this research; the CNC onset temperature was determined as being between 220 °C and 306 °C [15,16,20]. The temperature where maximum weight loss occurred (the DTGA temperature) was determined as 331.4 °C for CNF and 247.9 °C for CNC. In the beginning, it was thought that the DTGA temperature of CNC was lower than typically reported in the literature [15,16,20]. However, it was discovered that CNC has a second peak in the weight loss diagram, as shown in Figure 2b.

Wang et al. [21] and Kargarzadeh et al. [22] showed that acid hydrolyses play a significant role in the thermal stability behavior of CNCs, and they determined two processes (pyrolysis or peaks) in their studies (which were evident at 230 °C and 350 °C. Das et al. [16]; they also observed two different degradation peaks for crystalline cellulose, one at 250 °C and the second at 315 °C). The first peak can be related to the dehydration of cellulose to dehydrocellulose and the second peak can be related to the depolymerization of cellulose in competition with dehydration [23]. In another study on cellulose nanowhiskers (CNWs), it was reported that CNWs’ onset temperature changes from 177.5 °C to 224.5 °C, and the peak degradation temperature (DTGA) varies between 282 °C and 317 °C [24]. Cellulose nanocrystals show a gradual thermal transition, which occurs between 150 °C and 250 °C, with a 230 °C onset temperature [25,26].

As mentioned earlier in previous paragraphs, CNCs showed lower onset temperature and peak degradation temperatures compared to CNFs. Authors explain this with the expected higher surface area of CNCs, which result in greater exposure to heat. Also, the lower thermal stability of CNCs can be related to their higher crystallinity, which produces a fast heat transfer ability. Shimazaki et al. [27] reported that crystallized cellulose chains are efficient for pathways, which produces better thermal conductivity. In addition to the effect of crystallized structure, the introduced sulfate groups to the cellulose nanocrystals lower the thermal stability of CNCs [28]. In addition to sulfate groups on fibrils, particles surface through the hydrolysis step, reducing the activation energy for degradation and decreasing the level of resistancy to the pyrolysis [29].

The fibril and particle sizes are other important parameters for evaluating the thermal stabilities of the materials. The size effect plays an important role on having lower thermal stability. Compared to CNFs, CNCs have smaller sized, rice-shaped (whiskers) particles, which offer a higher surface area (that would allow higher sulfate introduction, which would lower the thermal stability) [30].

The residue of CNCs (34.4%) was significantly higher (50.9% higher) than the residue of CNFs (22.8%) at 500 °C. The literature studies showed similar results with this research; Lu and Hsieh [25] determined 70.3% weight loss (29.7% residue) from the cellulose nanocrystals at 600 °C; Fortunati et al. [26] found 23% residue at 500 °C for CNCs, that was produced from microcrystalline cellulose; and Rahimi et al. [31] compared CNF, CNC, and pure bagasse fibrils and found that acid hydrolysed CNC samples significantly have a higher residue compared to CNF samples. In another interesting study performed by Kargarzadeh et al. [18]; it was found that the residue varies between 27% and 44.8% for CNCs, which was related to hydrolyses time variations between 20 and 120 min. The samples acid hydrolysed for 20 min showed minimum char (27%), and the samples with 120 min hydrolyze time showed a higher amount of char (44.8%). This can be related to and supported with earlier studies, where it was shown that acid hydrolyses decrease particle sizes and increase the surface area available for sulphate introduction (and these sulfated groups may act as flame retardants [28] and increase the char yield). Also, the smaller particle sizes create increased the number of end chains, which decomposed at low temperatures [32] (which also provides an increase in char yield) [33].

## 8. Conclusions

The nanomechanical and thermal stability performances of the cellulose nanomaterials (CNs) (specifically, two different types: cellulose nanofibrils (CNFs) and cellulose nanocrystals (CNCs)) were determined and compared in this study. The nanoindentation technique, using two different approaches (the Oliver-Pharr and Fused Silica approaches) was performed to investigate CNs’ nanomechanical properties for increased accuracy. CNCs showed higher mechanical performance in both approaches, which can be related to CNCs’ potential higher crystalline structure (where the molecules are very well organized and capable of transferring the applied stresses smoothly, compared to CNF). Contrary to the positive effect of the crystallinity on the nanomechanical performance of cellulose nanomaterials, it lowered the thermal stability properties of CNCs. CNCs are introduced to a higher amount of sulfate groups through the production process, which produces a pathway that has a fast heat transfer capability. This pathway decreases the thermal degradation temperatures of CNCs. Also, higher surface areas of CNCs, exposed to the heat source, lower the thermal stability.

This research provides increased knowledge about the thermal stability and nanomoechanical performance properties of cellulose nanomaterials. Future work will include investigating the crystallinity and surface area of cellulose nanomaterials, and developing a relationship between their nanomechanical and thermal properties, theoretically.

## Figures and Tables

**Table 1:** Calibration test results.

Calibration Method	N (Number of Indents)	*H* (Contact Depth (nm))	*E* (GPa)
OP	64	66.6 (15.6)	128.9 (16.2) A
FS			69.7 (11.1) B

Parentheses indicate the coefficient of variation (COV, %). A and B letters indicates the significant differences (α = 0.05) between the OP and FS Approaches.

**Table 2:** The nanomechanical properties of CNF.

Calibration Method	N (Number of Indents)	CNF Modulus (GPa)	*H* (nm)
OP	64	15.1 (8.4) A	249 (6.5)
FS		12.4 (8.5) B	

Parentheses indicate the coefficient of variation (COV, %). A and B letters indicate the significant differences (α = 0.05) between the OP and FS approaches.

**Table 3:** The nanomechanical properties of CNC.

Calibration Method	N (Number of Indents)	CNC Modulus (GPa)	*H* (nm)
OP	64	28.7 (7.2) A	293 (3.1)
FS		21.1 (6.3) B	

Parentheses indicate the coefficient of variation (COV, %). A and B letters indicate significant differences (α = 0.05) between the OP and FS Approaches.

**Table 4:** The comparison of thermogravimetric properties of CNF and CNC.

Sample	N (Repetition)	Onset Temperature (°C)	DTGA Temperature (°C)	Residue (%)
CNC	5	228.2 (1.5) B	247.9 (0.2) B	22.8 (1.9) B
CNF	5	279.9 (1.5) A	331.4 (0.3) A	34.4 (1.6) A

Parentheses indicate the coefficient of variation (COV, %). A and B letters indicate significant differences (α = 0.05) between CNF and CNC.
